# The significance of N-carbamoylglutamate in ruminant production

**DOI:** 10.1186/s40104-023-00854-z

**Published:** 2023-04-13

**Authors:** Susan A. McCoard, David Pacheco

**Affiliations:** AgResearch Limited, Grasslands Research Centre, Tennent Drive, Private Bag 11008, Palmerston North, 4442 New Zealand

**Keywords:** Dairy, Environment, Fetal growth and development, Lactation, N-carbamoylglutamate, Nitrogen, Red meat, Reproduction

## Abstract

Improving the efficiency and production of grazing ruminants to support food and fiber production, while reducing the environmental footprint and meeting the welfare needs of the animals, is important for sustainable livestock production systems. Development of new technologies that can improve the efficiency of nitrogen (N) utilization in ruminants, and that are effective and safe, has important implications for ruminant livestock production. N-carbomoylglutamate (NCG) is a functional micronutrient that stimulates endogenous synthesis of arginine, which can improve survival, growth, lactation, reproductive performance, and feed efficiency in mammals. There is a growing body of evidence to support the potential of dietary NCG supplementation to improve the productive capacity and N utilization efficiency of ruminants. This review summarizes the current literature on the effects of dietary supplementation with NCG in ruminants and impacts on production and potential to reduce the environmental footprint of farmed ruminant livestock. The current literature highlights the potential for commercial application in ruminant livestock to improve productivity and N utilization efficiency.

## Introduction

Global production of animal products is predicted to nearly double by 2050 as human population and incomes rise [1]. Of the 51 million km^2^ of land used for agriculture globally, 77% (40 million km^2^) is used for livestock production, which contributes 18% and 37% of global food energy intake and food protein supply, respectively [2]. In temperate climates, pasture-based ruminant livestock production systems offer a lower-cost diet than grain-based diets, and sustainable farming systems to produce high quality food for human consumption [3, 4]. Improving the efficiency and production of grazing ruminants to support the production of food and fiber, while reducing the environmental footprint and meeting the welfare needs of the animals is important. Therefore, the development of new technologies that can improve the efficiency and production of grazing ruminants, and that are effective and safe, has important implications for ruminant livestock production.

Feeding practices are an important determinant of the efficiency of livestock production and are a climate change adaptation measure [5]. Shifting feed resources from forage to grain-based diets has improved efficiency in beef and dairy industries to meet the increasing nutritional demands of improved animal genetics [6]. However, feeding grain to livestock competes with the human food chain. Production of grain is expensive and importation of grain for both human and livestock feed is required in some countries, thereby increasing the carbon footprint of this food source [3]. Ruminants have the advantage of converting roughage and non-protein N sources that cannot be digested by humans, into food (e.g., milk, meat) and co-products (e.g., wool) [7]. However, ruminants are unable to efficiently utilize all the N in feed, with approximately 70% of ingested N being excreted in faeces and urine [8, 9]. This results in losses in production efficiency, and contributes to environmental pollution. Improving feed conversion is a critical contributor to lowering the carbon footprint of food as 70%–90% of the emissions in the total chain occur within the farm gate [10].

## Metabolic requirements for amino acids

Nitrogen is a key structural component of amino acids (AA). In all animals, AA are building blocks for proteins and are essential substrates for the synthesis of many physiologically important substances including nitric oxide (NO), polyamines, glutathione, creatine, carnitine, carnosine, thyroid hormones, serotonin, melatonin, and heme [11]. Nutritional studies have also demonstrated that several nutritionally non-essential AA (NEAA, e.g., arginine, glutamine, glutamate, leucine and proline) play important roles in regulating gene expression, cell signaling, antioxidative responses, fertility, neurotransmission and immunity [11]. These findings led to the concept of functional AA, which is defined by Wu [12] as “those AA that participate in and regulate key metabolic pathways to support health, survival, growth, development, lactation and reproduction of organisms”. Supplying these AA in the diet may supplement the endogenous synthesis and thus support survival, growth, development, reproduction, and health. These findings also highlight the potential to improve the efficiency of nutrient utilization, growth, and production performance of livestock through modifications in dietary AA, and has elicited a rethink of the dietary requirements for all AA by livestock species [13]. This is particularly important in pasture-based grazing production systems for ruminants as fresh forages and crops contain a large proportion of ruminally degradable protein and therefore microbial crude protein becomes the main source of AA for the animal.

## Arginine and arginine precursors

Arginine (Arg) has a central role in N metabolism, blood flow, nutrient utilization, health, and production (e.g., milk, reproduction, growth) of ruminants [14]. Arginine also has a well-recognized function in ureagenesis and ammonia detoxification [15]. Evidence indicates that maximal growth and production performance of pre-ruminants, gestating and lactating sheep and cows require dietary Arg [16] and that dietary supplementation of rumen protected Arg or stimulating endogenous Arg synthesis through Arg precursors can improve these production indices without adverse metabolic or health effects [14]. Thus, there is an important role for Arg nutrition in sustaining ruminant production worldwide [14, 17, 18].

It is not possible to raise circulating concentrations of Arg in ruminants through oral supplementation as it is rapidly degraded in the rumen [14]. Parenteral administration, i.e., direct via the bloodstream, to farm animals is not a practical option, and rumen-protected formulations that are affordable and effective are yet to be developed. The biological half-life of Arg is also relatively short, 45 min in sheep and sows [15, 19] because of high arginase activity causing rapid degradation of this AA in tissues [20, 21]. Other compounds that are involved in the urea cycle and/or increase Arg synthesis have therefore been investigated including citrulline [14, 22] and N-carbamoylglutamate (NCG; also known as N-carbamylglutamate). NCG is a substantially cheaper alternative to Arg [23] and is a more effective Arg precursor than citrulline, at least when supplemented to animals during gestation [22]. Chacher et al. [24] reviewed the role of NCG in the biosynthesis of Arg and speculated on its potential application as a supplement to improve the production of ruminant animals. However, supporting literature from ruminant studies was limited at that time. A subsequent review by Palencia et al. [22] reported a systematic review of the literature on the effectiveness of NCG on mammalian reproductive performance, reviewing data from 8 studies, of which only 4 were in ruminants (sheep). There has been recent growth in the literature on the potential role of NCG in ruminant livestock production, which is the focus of the present review.

## NCG action and safety

**Fig. 1:**
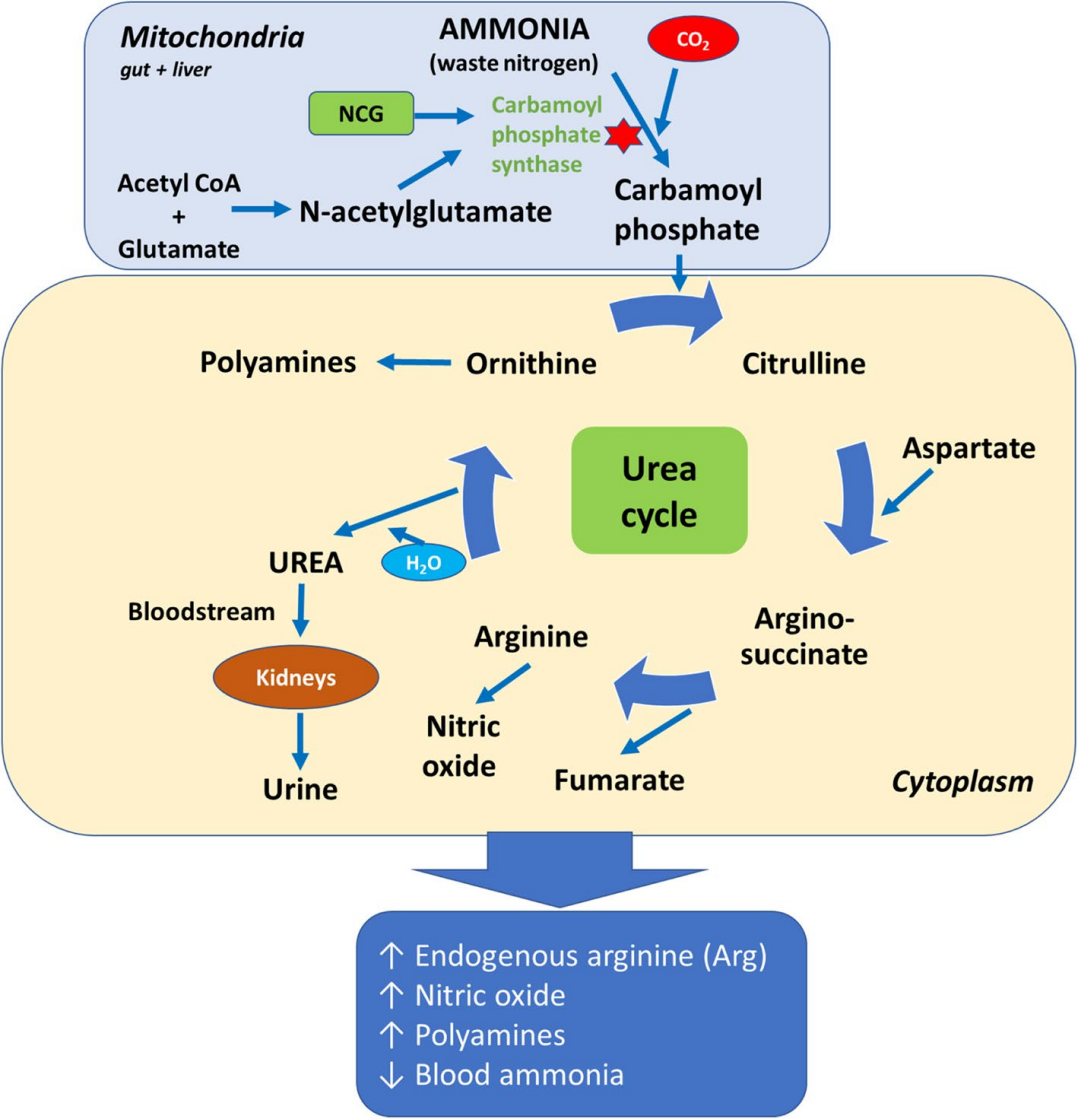
A schematic to illustrate the influence of N-carbomylglutamate (NCG) on carbamoyl-phosphate synthase, the rate limiting enzyme of the urea cycle pathway, and downstream effects on the urea cycle pathway in ruminants

N-acetyl glutamate (NAG) is an endogenous AA derivative, which increases the endogenous synthesis of Arg by activating carbamoyl-phosphate synthetase-1 (CPS-1), a key enzyme in the urea cycle pathway, in the mitochondria of hepatocytes and intestinal cells [23]. NAG is easily hydrolyzed however, making it impractical to use therapeutically as it is degraded in the rumen. NCG is a metabolically stable structural analogue of NAG [15, 20, 25]. NCG is more resistant than NAG to hydrolysis by aminoacetylase. Degradation of NCG in rumen fluid over 24 h is 17.8%, compared to 100% degradation of Arg [26]. This enables NCG to pass into the small intestine where it activates the urea cycle pathway to produce Arg, NO and polyamines in both intestinal enterocytes as well as in the liver following absorption (Fig. 1).

NCG has no cellular action other than serving as an activator of CPS-1 and intestinal pyrroline-5-carboxylate synthase. Therefore its pharmacodynamic effects are limited to this action on the urea cycle. No off-target activity has been observed in any toxicity studies [27]. Administration of NCG does not alter the intestinal availability of intestinal ammonia or other nitrogenous precursors needed by gut microbes, nor affects intestinal absorption of other diet-derived AA (e.g., tyrosine, histidine, lysine) [23]. Toxicity studies have been undertaken in various species indicating low toxicity with the “No Observed Adverse Effect Level” (NOAEL) for oral administration in rats being 2806 mg/kg (https://www.ema.europa.eu.). Eco-toxicity (persistence in water/soil, bioaccumulation, and soil mobility) is also low (CAS number 1188–38-1, Chemwatch MSDS). Wu et al. [28] also demonstrated that NCG is a non-toxic substance with no genotoxicity and therefore is safe for both humans and animals. Based on predictive modelling using BioTransformer (http://biotransformer.ca/), there is no Phase 1 metabolism of NCG and only limited Phase II metabolism with the production of glucuronide to excrete via urine and carbon dioxide (Karl Fraser, personal communication). NCG has now been widely used in many monogastric and ruminant species [22] and the present review with no reported adverse effects. We have demonstrated target animal safety in sheep with no adverse effects observed for any physiological traits in either 1 (150 mg/kg BW), 3 (450 mg/kg BW) or 5 (750 mg/kg BW) times dose rate groups (McCoard et al., unpublished observations). Similarly, NCG is widely used in humans, e.g., for treatment of certain rare diseases associated with ammonia detoxification [29, 30] at similar dose rates as those used in animals, and for repeated use over the lifetime with no reported serious adverse effects. NCG is not an antibiotic, as it did not kill microbes isolated from the small and large intestine of 7–21-day old pigs [23] nor rumen microbes in vitro [26]. Therefore, the potential risk profile for use of this compound in food producing animals is considered low.

## NCG and ruminant lactation performance

Milk is an essential nutrient for the growth, development, and health of mammalian neonates. Milk production is a limiting factor for pre-weaning growth performance and survival of offspring in livestock species including ruminants [31]. Colostrum is the first secretion from the mammary gland and is essential for passive transfer of immunity to the neonate to support the underdeveloped neonatal immune system [32]. Colostrum and mature milk also contain oligosaccharides [33] and non-nutrient substances (e.g., insulin-like growth factor I) and other bioactives that are essential for growth, development, health, and survival of neonates [34]. Milk from livestock species also has high nutritional value for humans of all ages [35]. Therefore, opportunities to optimize lactation performance in ruminants is important for both the nutrition of young livestock as well as human nutrition.

Amino acids play an important role in the development and function of the mammary gland, which has important implications for both milk production and neonatal growth [17, 31]. For example, post-ruminal supply of Arg (0.1 g/kg liveweight) via the jugular vein once daily in the last 7 d of gestation in Holstein dairy cows tended to increase milk production (~ 10%) during the first 22 weeks of lactation [36]. Continuous parenteral supply of Arg (9.42 g/L *L*-Arg) via the jugular vein for 1 week during early lactation (20 ± 2 d in milk) also increased milk yield, milk protein yield and milk efficiency (milk yield/feed intake) in Chinese Holstein dairy cows [37]. In sheep, parenteral supplementation with Arg (345 μmol/kg bodyweight weight via intravenous thrice daily) from d 100 to term in Romney sheep was associated with increased mammary gland cellular content prior to parturition, reduced milk somatic cell count (SCC) and a transient increase in milk protein yield, and the absolute concentration of some milk free AA, while milk yield was unaffected [38]. These observations highlight the potential for arginine to improve milk production.

The effect of dietary NCG supplementation on milk production traits in ruminants has received recent attention. Four studies in high-yielding Chinese Holstein dairy cows housed indoors, fed a total mixed ration (TMR) diet, and milked twice daily, and one study in lactating cross-bred Boer × Yangtse River Delta White goats have been undertaken in China, each at different physiological stages of gestation or lactation (Table 1). When supplemented in the diet in the last 4 weeks of gestation and first 10 weeks of lactation, NCG increased milk yield, milk protein and milk fat yield and percentage while milk SCC and feed efficiency were unaffected in high-yielding Chinese Holstein dairy cows [42]. These authors suggested that these effects may have been mediated via increasing circulating concentrations of Arg and NO and improving AA supply and utilization and/or liver function and potentially feed efficiency.

**Table 1:** Summary of published studies evaluating the effect of N-carbamoylglutamate supplementation to ruminants on lactation performance

Animals	Dose	Delivery method	Duration	Outcome	Reference
60 Chinese Holstein multiparous dairy cows; 78 ± 17 DIM, 635 ± 60 kg BW and 42 ± 8 kg/d milk yield	0, 10, 20, 30 g/NCG/d; 50:50 mix with corn starch	Top dressed on TMR (17.6% CP) twice daily	10 d adaptation and 7 weeks treatment (3 times daily milking)	↑ Milk yield (2 kg/d) – trend in 20 g/d ↑ Milk protein content (2.8% vs. 2.7%) and yield (1.12 vs. 1.02 kg/d) in 20 g/d ↑ Lactose and milk solids (linear ↑ with NCG dose) No change DMI, milk fat %/yield	[39]
60 Mid-lactation Holstein dairy cows 176 DIM 669 kg BW, milk yield 26 kg/d	0, 10, 20 or 40 g/hd/d NCG	Top dressed on TMR (13.9% CP) 3 times daily 0600, 1400 and 2000 h	2-week adaptation, 12 weeks treatment	↑ Fat yield (linear; 0.97 vs. 0.91, 40 g/d) ↑ Fat % (linear and quadratic; 4.24 vs. 3.93, 40 g/d) ↑ Protein % (linear; 3.74 vs. 3.62, 40 g/d) ↑ Total solids (linear; 13.3 vs. 13.0, 40 g/d) ↑ Lactation persistency (linear and quadratic; 99.4% vs. 89.2%, 20 g/d) No change DMI or milk, protein, lactose, fat, energy corrected milk yield or feed efficiency	[40]
Subset of 30 cows from 0 and 40 g/d group from [40]	0 vs. 40 g/NCG/cow/d	Top dressed on TMR 3 times daily 0600, 1400 and 2000 h	8 weeks	↑ Milk NCG concentration (6 times) No NCG in cheese in control, < 1.0 μg/kg in NCG group (in whey stretch water, brine during cheese production) No effect on cheese texture and colour but NCG group lower in hardness	[41]
30 Chinese Holstein dairy cows- multiparous; individual tie stalls; BCS 3.39 (5-point scale), 657 kg BW, 2.73 parity, 8692 kg 305d milk yield	0 and 20 g/d NCG	Top dressed on TMR (12.6% CP dry period and 17.3% CP lactation) 1 time daily at 14:00	4 weeks pre-calving to 10 weeks post-calving	↑ Milk yield (40.9 vs. 37.7 kg/d) ↑ Protein yield (1.2 vs. 1.13 kg/d) ↑ Fat yield (1.74 vs. 1.55 kg/d) Trend for ↑ fat, milk solids and lactose % over time Trend for ↓ protein % over time ↑ Liver function No change feed conversion efficiency No change SCC – numerical ↓ (3.82 vs. 4.2, *P* = 0.12)	[42]
48 heat stressed Holstein lactating dairy cows; 154 DIM, 1–3 parity	0, 15, 20, 25 g NCG/d	Mixed in TMR (CP not reported) diet – frequency not stated (milked 3 times daily)	60 d	↑ Milk yield (linear and quadratic; 31.5 vs. 29.9 kg/d) ↑ Protein % (3.49 vs. 3.27) ↓ SCC (3.33 vs. 4.65 × 10^4^/mL, 20 g/d best) ↓ Pulmonary hypertension Improved immune function and antioxidant capacity No change lactose %, fat %, DMI	[43]
14 Jersey cows 385 ± 46 kg body weight	0, 20 g NCG/d	Mixed in ad libitum TMR diet (8.8% CP)	60 d	↑ DMI (trend) ↑ Blood oxygen saturation (trend) ↑ Milk fat % (trend) ↓ Milk protein % (trend) ↓ Markers of high-altitude stress ↓ Molar proportion of butyric acid (trend) but no other change in rumen fermentation No change in nutrient digestibility, plasma immunity and antioxidant capacity Altered lipid and amino acid metabolism	[44]
Lactating cross-bred Boer × Yangtse River Delta White goats with twin male suckling kids	0, 1, 2 or 3 g/d/goat	Mixed in TMR diet (15.9% CP) – frequency not stated	0–42 d lactation	↑ Milk yield at 21 (6.12 vs. 5.12 kg) and 42 d (8.62 vs. 6.99) in 2 g/d group only ↑ Milk protein % (2 g/d group only)	[45]

When NCG was supplemented via the diet in early lactation (61–95 d in milk) for 7 weeks, a trend to increase milk yield and an increase in the concentration and yield of milk protein (but not fat) were observed but only when cows were supplemented with 20 g/d (~ 30 mg/kg BW) compared to controls, with no effects on milk production observed in the 10 or 30 g/d supplemented groups [39]. In that study, plasma Arg concentrations were increased by 1.1%, 10.4% and 16.0% for the 10, 20 and 30 g/NCG/d groups respectively, highlighting a dose–response effect of NCG supplementation in early lactation on plasma Arg concentrations, while milk production was only increased in the 20 g/d NCG supplemented group. Similarly, in crossbred Boer × Yangtse River Delta White goats supplemented with 1, 2 or 3 g/d from d 0–42 of lactation, compared to controls (0 g/d), circulating Arg concentrations were increased 24% in the 1 g/d group and by ~ 80% in the 2 and 3 g/d groups while milk yield was only increased in the 2 g/d NCG group [45].

In mid-lactation, when NCG was supplemented in the diet for 12 weeks (10, 20 or 40 g NCG/d; approximately 15, 30 or 60 mg/kg body weight) compared to untreated controls, linear increases in fat yield and concentration, protein concentration and total solids were observed but only the 40 g/d NCG group was significantly different to controls [40]. Linear and quadratic improvements in lactation persistency were reported in the 20 g/d NCG group relative to controls [40]. No other effects on dry matter intake, milk yield, protein, lactose, fat- corrected milk yield, or feed efficiency were reported [40]. When heat-stressed Holstein dairy cows were supplemented in mid-lactation with 0, 15, 20 or 25 g NCG/d for 60 d, milk yield was increased in all groups, but no dose–response effect was observed. Milk protein concentration was increased in the 15 g/d group only compared to all other groups while milk fat, lactose percentage and milk SCC were unaffected [43]. Dietary NCG supplementation to Jersey cattle in high altitude areas may also improve milk quality and prevent hypoxic stress associated with high altitude [44].

Collectively, the aforementioned studies indicate that NCG supplementation to dairy cows has variable effects on milk yield and composition depending on the dose rate and physiological stage in which supplementation was applied. This is likely due to the substantial changes in the physiological requirements for AA and thus AA metabolism during late gestation and early lactation relative to mid-late lactation, as well as the composition of the base diet (e.g., CP, AA composition etc.). The mammary gland undergoes considerable development during late gestation and early lactation to support colostrum and milk production which may influence the response of animals to dietary supplementation with NCG. These studies highlight that in addition to dose rate, the physiological and metabolic variations due to stage of the reproductive cycle (e.g., gestation vs. lactation) and/or lactation stage (e.g., early vs. mid-lactation) and/or environmental stressors (e.g., heat stress) and/or diet macro- and/or micro-nutrient composition may influence the lactation response of dairy cattle to NCG supplementation.

Overall, these studies indicate that there is potential for NCG supplementation to benefit lactation performance in ruminants. However, further studies are required to determine dose–response effects of NCG supplementation at different physiological stages, as well as to evaluate the effect of other potential factors that may influence AA metabolism and supply and therefore the response to NCG supplementation. These may include species, breed, level of milk production (e.g., low vs. high yielding animals), diet type (e.g., pasture vs. total mixed ration), allowance and composition (e.g., macro- and micro-nutrient content), and other environmental stressors (e.g., heat and cold stress, litter size, nutritional status).

## NCG impact on milk quality, composition and cheese production in ruminants

In addition to milk being critically important in supporting neonatal survival, growth and health, milk and cheese are important sources of nutrition for humans, serving as a good source of protein and delivery of many bioactive compounds [46]. Thus, health benefits and quality of milk and cheese have been the focus of many studies [47]. Milk quality and cheese flavour can also be influenced by milk AA composition [48]. Cheese is also a popular food source and a healthy snack for children [49].

Using a subset of animals from the mid-lactation NCG supplementation study [40], Gu et al. [41] reported that cows supplemented with 40 g NCG/d compared to unsupplemented controls increased NCG levels in milk sixfold relative to controls (6.6 vs. 1.1 µg/L) with two thirds of the NCG being present in the whey fraction with the remainder in the cheese, stretch water and brine. Small amounts (< 1.0 µg/kg) of NCG were detected in cheese from the treated cows and no NCG was detected in the cheese from control animals. While cheese was softer when made from milk of NCG treated cows, no differences in cheese texture and colour were observed. The concentration of both branched chain and NEAA was also increased in NCG-supplemented compared to control animals. These results illustrate that NCG supplementation can increase the AA concentration of raw milk but does not affect cheese (mozzarella) quality. Further studies are required to determine whether altering the level of dietary NCG supplementation, supplementation during different stages of the lactation cycle, differences in basal diet and/or species-specific differences influence the quality of mozzarella and other types of cheeses.

The potential benefits of NCG in milk for human health were postulated by Gu et al. [41]. This notion was based on the growing number of studies that suggest NCG supplementation in animals could improve liver function and intestinal health and reduce oxidative stress [50–52]. In humans, NCG is also used to treat hyperammonemia in rare inherited disorders [53, 54]. Such beneficial effects are likely the result of NCG stimulation of endogenous synthesis of Arg [23, 55] which has many biological functions in reproduction, immunity, neonatal growth and wound healing in both humans and other monogastrics [15]. Arg is also the precursor of NO which can regulate dilation of blood vessels, angiogenesis and increase blood flow [56]. Arg and NO can also relax blood vessels and prevent cardiovascular disease [57, 58]. Therefore, NCG transferred into milk may confer some health benefits to humans and/or animals that consume milk from supplemented animals. Additionally, the increase in AA concentration of milk, in particular AA such as Arg, may also have beneficial effects on human and animal health, nutrition, and wellbeing alike.

## NCG and ruminant reproduction

An in vitro study using bovine granulosa cells indicated that both NCG and Arg may influence ovarian function by acting directly on these cells to slow follicular differentiation by stimulating their proliferation and inhibiting insulin-like growth factor-1 (IGF-1) action and steroid synthesis [59]. A recent study by Zhou et al. [60] reported that supplementing multiparous anoestrous female yaks with 0 or 6 g NCG/d promoted follicular development through modulation of cholesterol metabolism to initiate steroidogenesis in the ovaries of yaks [60]. While there is some evidence that Arg plays an important role in the development of reproductive organs in both males and females, there is a paucity of data in ruminants [14, 17]. Further research is required to determine the potential role for NCG to influence reproductive physiology, critical time windows for intervention and production efficiency impacts in ruminants. This is particularly important for dairy cows fed diets rich in crude protein (CP) which has been associated with decreased fertility [61]. The ability of NCG supplementation to increase conception rates in cows fed high-protein diets has been hypothesized previously [24] but this has not yet been evaluated directly. Further studies are required to evaluate the effect of NCG supplementation on fertility and reproductive success in both small and large ruminant species. Such studies must be statistically powered to understand both the underpinning biology as well as the commercial impacts.

It is well established that Arg plays a key role in fetal growth and development in ruminants [14, 17]. There is also a growing body of evidence to suggest that NCG supplementation can, through its action on endogenous arginine production, also enhance aspects of fetal growth and development (Table 2). Dietary supplementation of Inner Mongolia white Cashmere goats with NCG at 0, 0.3 or 0.4 g/d for the first 90 d of gestation increased arginine concentrations in the maternal plasma in a dose–response manner, but fetal body, heart and liver weight and brown adipose tissue mass at 90 d gestation was only increased in the 0.4 g/d group relative to controls [67]. Supplementation of undernourished (50% of NRC requirements) twin-bearing Hu ewes from d 35 to 110 of gestation with 5 g/d NCG mixed into a TMR diet increased fetal growth at 110 d of gestation compared to undernourished control ewes but did not fully ameliorate the effects of undernutrition relative to ewes fed 100% of NRC requirements [69]. Improved fetal growth was postulated to be mediated at least in part through improved metabolic homeostasis, maternal–fetal-placental antioxidant capacity and placental angiogenesis. In that study, placental weight, development and efficiency was unaffected, however some aspects of placental angiogenesis were positively influenced by NCG supplementation which may have improved placental nutrient transfer [70, 71]. In the fetuses of the supplemented ewes from the study undertaken by Zhang et al. [70], improvements in fetal intestinal AA profiles were reported [73] along with improved fetal thymus development and immune function. However, the effects of NCG supplementation to ewes and their fetus(es) after d 110 of gestation to term, which is the physiological time period during which conceptus nutrient demand is the highest [74], has not been evaluated. Furthermore, the impacts on the ewe and lamb(s) post-birth have not been evaluated to date in sheep.

**Table 2:** Summary of published studies evaluating the effect of N-carbamoylglutamate supplementation to ruminants on reproduction

Animals	Dose	Delivery method	Duration	Outcome	Reference
Suckling Hu lambs (IUGR twins, 7 d old)	1% Arg or 0.1% NCG	In milk replacer	7–28 d	↑ Body weight, serum insulin and ↓ cortisol; mitigated negative effect of IUGR on nutrient absorption ↓ IUGR-induced apoptosis of hepatic cells ↑ Expression of antioxidative enzymes, phase II metabolizing enzymes and activation of the NO pathway to help protect from IUGR-induced hepatic oxidative damage ↑ Hepatic energy status and mitochondrial function ↑ Duodenal barrier function and mitochondrial function, mitigated duodenal inflammation and suppressed mitophagy ↑ Energy status, motivated key enzyme activities in the TCA cycle, inhibited AMP-activated protein kinase signalling pathway and ameliorated intestinal injury ↑ Intestinal function by regulating the oxidant status via the NO-dependent pathway ↑ Growth, intestinal integrity, immune function and oxidative status	[52, 62–66]
Lactating cross-bred Boer × Yangtse River Delta White goats with twin male suckling kids	0, 1, 2 or 3 g/d/goat	Mixed in TMR diet (15.9% CP) – frequency not stated	0–42 d lactation	↑ ADG and some aspects of rumen development of kids pre-weaning	[45]
48 Inner Mongolia white cashmere goats	0.3 or 0.4 g/d	Mixed with basal TMR diet	d 0–90 of gestation	↑ Arg family of amino acids and glucogenic amino acid concentrations in blood ↑ mRNA expression of osteopontin, αv and β3 integrins and endometrial development ↑ Fetal brown adipose tissue stores and UCP-1 and BMP-7 expression	[67]
32 Hu twin-bearing Hu ewes	Control (100% NRC) 50% NRC + 50% mineral-vitamin mix 50% NRC + 20 g RP-Arg + 50% min-vit mix 50% NRC + 5 g/d NCG + 50% min-vit mix	Mixed in basal diet	35–110 d gestation	No effect of RP-Arg or NCG on placental weight, development, or efficiency. Some changes in gene expression in placentomes (VEGFER2 but not VEGFA, VEGFR1, PLGF1, ANG1/2 Altered metabolome profiles ↑ Fetal growth to intermediate between 50% NRC and 100% NRC (another paper claims ↑ to 100% NRC group) ↑ Plasma AA—improved metabolic homeostasis↑ maternal–fetal-placental antioxidation capability and promote fetal growth (intermediate between 50% NRC and 100% NRC) and placental development with RP Arg and NCG in underfed ewes ↑ AA availability in the conceptus to improve fetal growth in underfed ewes ↑ Immune function and thymus development in IUGR fetuses	[68–71]
Bovine granulosa cells (in vitro)	0, 2 and 4 mmol/L	Serum-free medium for 24 h to 48 h	In vitro	Inhibited IGF1-, progesterone- and FSH-Induced granulosa cell estradiol production Inhibited StAR, CYP11A1 and CYP19A1 mRNA abundance in small-follicle granulosa cells ↑ Granulosa cell numbers induced by IGF1 and FSH Suggested direct effect on granulosa cells to regulate ovarian function by slowing follicular differentiation via inhibiting IGF-1 action, and steroid synthesis while stimulating granulosa cell proliferation	[59]
Yak ovaries	6 g/d/head	Mixed into TMR diet	d 0–32 gestation	↑ Large follicle (> 5 mm diameter) number ↑ Angiogenesis and de novo cholesterol synthesis in ovaries ↑ Steroidogenic enzyme expression in ovaries Suggested NCG promotes follicular development by influencing cholesterol metabolism to initiate steroidogenesis in ovaries	[60]
Multiparous Chinese Holstein dairy cows	0 or 20 g NCG/d/head (*n* = 15/group)	Top dressed on TMR (12.6% CP dry period)	Last 28 d of gestation	↑ Calf birthweight (5 kg) ↑ Plasma Arg in neonatal calves Altered markers of placental angiogenesis and uteroplacental nutrient transport ↑ AA metabolism and urea cycle of the calf	[72]

Gu et al. [72] reported that dietary supplementation of multiparous Chinese Holstein dairy cows in the last 28 d of gestation increased newborn calf weight by ~ 5 kg which may have been mediated at least in part by enhanced placental expression of mTOR (a key regulator of nutrient transport) and angiogenic factors to enhance nutrient supply to the fetus. Supplementation with NCG from 0–90 d gestation in goats increases circulating Arg concentrations and may improve brown adipose tissue development [67]. 

Collectively, these studies suggest that maternal supplementation with NCG during gestation has the potential to be beneficial for fetal growth and development in ruminants. However, further studies are required in both small and large ruminant species to establish dose–response effects on the dam (e.g., health and lactation performance), as well as the progeny both in utero and during postnatal life (e.g., survival, growth, reproductive performance). Furthermore, critical time windows for supplementation, as well as potential species and breed differences and other environmental factors such as dietary factors (e.g., nutritional status, diet type, allowance, and composition), litter size, and climatic conditions need to be evaluated. Such studies are required to determine the potential application of gestational supplementation of NCG to livestock species.

There is some emerging evidence that NCG supplementation of milk replacer can support growth and development of growth-restricted lambs or kids. Supplementing milk replacer with 0.1% NCG from d 7 to 28 of neonatal life to intra-uterine growth restricted (IUGR) lambs increased their growth rate and improved aspects of hepatic, intestinal and immune function, compared to unsupplemented IUGR lambs [62–66, 68] but not non-IUGR lambs. In suckling goat kids, NCG supplementation of milk replacer with 100 mg/kg body weight from 1 to 41 days of age increased growth rates and weights of some organs (e.g., spleen) with corresponding increases in the concentration of Arg family AAs in the blood, reduced the incidence of diarrhoea and reduced plasma ammonia and urea N concentrations suggesting a reduction in catabolism of AA, and/or an improvement in the capture of ammonia N in the rumen by microbes [66]. Wang et al. [45] also reported increased growth rates of kids that were reared by dams supplemented with 2 or 3 g/d of NCG in their basal TMR diet however the mechanisms involved have not been determined. These studies suggest potential for NCG supplementation to improve the growth and development of neonatal ruminants when either supplemented via milk replacer in artificial rearing systems, or via supplementation to the dam. Further studies are required to determine both the short (pre-weaning) and long-term (post-weaning) effects of neonatal NCG supplementation via milk replacer on neonates that have not been subject to in utero growth restriction. Furthermore, potential species and/or breed effects, critical time windows for supplementation, and potential influence of other environmental factors such as birth weight, litter size, and composition and allowance of milk replacer have not been investigated.

## NCG effects on growth and red meat production in ruminants

Globally, red meat production is important for the generation of a high-nutritional food source for humans, as well as employment. New technologies to support sustainable improvements in on-farm efficiency and productivity are required to meet future challenges such as climate change, consumer acceptance of livestock farming, changes in consumer preferences and land-use changes [4, 17]. The effects of NCG on N metabolism in beef cattle has been investigated in 2 studies in China (Table 3). Yang et al. [75] reported that supplementation of fattening Holstein bulls (408 kg body weight and 450 days of age) supplemented with 0, 20, 40 or 80 mg/kg body weight of NCG/d fed in a 11.3% CP grain-based diet for 7 weeks increased average daily gain (ADG), feed conversion efficiency, N retention and N utilization. A reduction in faecal and urinary N (UN) concentration in NCG-supplemented bulls was also reported. However, this finding was based on a subsample pooled from each animal on specific trial days and using a marker for digestibility and urinary volume rather than total collection of excreta. Further studies are required where total collection of urine and feces per animal is undertaken along with feed intake, diet composition and greater liveweight and lean tissue mass in the carcass in order to confirm differences in N retention in response to NCG supplementation. Zhang et al. [76] reported that relative to a control diet, NCG supplementation with 40 mg/kg body weight/d to a grain-based control diet with urea (13.5% CP) or without (12.8% CP), increased ADG, feed conversion rate, digestibility of dry matter and CP, without changing feed intake. Faecal and UN concentration were also reduced, and N utilization increased while plasma ammonia also decreased with a similar trend in plasma urea concentrations. Slaughter weight was unaffected by NCG supplementation, but carcass weight was increased.

**Table 3:** Summary of published studies evaluating the effect of N-carbamoylglutamate supplementation to ruminants on growth and red meat production

Animals	Dose	Delivery method	Duration	Outcome	Reference
Cattle (Holstein bulls; 408 kg 450 d old); *n* = 24 (6/group)	0, 20, 40, 80 mg NCG/kg BW	Mixed in feed		↑ ADG, FCR, CP digestibility, N retention, N utilization (linear + quadratic) ↓ Fecal and urinary N (linear) ↓ Plasma ammonia and urea	[75]
Holstein bulls [490 kg BW; *n* = 24 (6/group in individual pens)]	Control diet (0) vs. Control diet + NCG (40 mg/kg BW) vs. urea diet vs. urea diet + NCG (40 mg/kg BW)	In diet	2-week diet adaptation 7-week treatment (2 times daily feeding)	Control vs. Control + NCG: ↑ ADG (1.48 vs. 1.74 kg/d) ↑ FCR (8.40 vs. 6.91 kg DMI/kg ADG) ↑ Carcass weight (357 vs. 381 kg) ↑ Dressing % (trend; 55.3 vs. 57.9) ↑ Eye muscle area and shear force ↓ Drip loss, Color L*, cooking loss %	[76]

The aforementioned studies suggest that there is potential utility for NCG supplementation to the diet to improve beef production. However, further studies are required to determine the potential dose–response benefits of NCG supplementation in meat producing animals of different species and breeds, different physiological states (e.g., pre- vs. post-weaning or finishing) as well as the potential interaction with diet including feeding levels, diet type (e.g., grain vs. forage) and macro/micro-nutrient composition, as well as environmental conditions which can influence nutrient requirements (e.g. indoor vs. outdoors, cold vs. hot climates). Studies are also required to determine animal and food safety attributes to establish the suitability of NCG to be used in food producing animals.

## Mitigation of nitrogen pollution

Improving N capture into milk and meat proteins in ruminants has benefits for improving production efficiency and could help reduce environmental N pollution via reductions in urine and faecal N excretion [77]. This is particularly important in temperate grazing ruminant production systems, where intensively-managed improved pastures (e.g., rotational grazing systems with N fertilizer input) often contain protein concentrations that exceed the nutritional requirements of ruminants [78], exacerbating the low efficiency of conversion of dietary N to milk/meat N [77]. Excretion of N to the environment is a significant issue for the sustainability of pastoral systems because excreta N, particularly in urine, contributes to the greenhouse gas footprint (nitrous oxide) and water pollution (nitrate leaching) in those systems [79]. Therefore, technologies that offer the potential to improve N utilization by the animal may contribute to increasing animal production performance while reducing environmental impact.

A summary of published studies evaluating the effect of NCG supplementation to ruminants that relate to the potential to mitigate environmental N pollution is provided in Table 4. When NCG is supplemented to ruminants, a reduction in plasma urea N (PUN) concentration has been commonly observed [39, 42, 43, 45, 52, 75, 76]. In some of these studies, the reduction in PUN concentration has also been accompanied by a reduction in milk urea N (MUN) concentration in lactating animals. In these studies, the changes in PUN and MUN have mostly occurred without N intake being affected, suggesting that NCG supplementation elicits changes in N metabolism that are not intake-driven. Reduction in PUN could be attributed to a reduction in AA deamination [80] thus sparing of AA for the protein synthesis underpinning productive purposes. Indeed, NCG has been reported to increase milk protein yield in cows [39, 42], goats [45] and N retention in bulls [73].

**Table 4:** Summary of published studies evaluating the effect of N-carbamoylglutamate supplementation to ruminants on the potential to mitigate environmental nitrogen pollution

Animals	Dose	Delivery method	Duration	Outcome	Reference
60 Chinese Holstein multiparous dairy cows; 78 ± 17 DMI, 635 ± 60 kg BW and 42 ± 8 kg/d milk yield	0, 10, 20, 30 g/NCG/d; 50:50 mix with corn starch	Top dressed on TMR twice daily	10 d adaptation and 7 weeks treatment (3 times daily milking)	↓ Plasma ammonia N (1200 vs. 595 μmol/L) ↓ MUN (12.4 vs. 11.5 mg/dL) ↓ BUN (7.06 vs. 6.24 mg/dL) ↓ UN (1071 vs. 794 mg/dL Greatest ↓ in 20 g/d group vs. control	[39]
Cattle (Holstein bulls; 408 kg 450 d old); *n* = 24 (6/group)	0, 20, 40, 80 mg NCG/kg BW	Mixed in feed		Best in 40 g/d group: ↓ BUN (2.54 vs. 1.99 mmol/L) ↓ Plasma ammonia (64.8 vs. 34.4 μmol/L) ↓ (trend) faecal N (70.5 vs. 62 g/d) ↓ (trend) urinary N (80.2 vs. 68.3) ↑ N retention (46.6 vs. 61.5 g/d) ↑ N utilization % (23.6 vs. 32.1)	[75]
Holstein bulls (490 kg BW; *n* = 24 (6/group in individual pens)	Control diet (0) vs. Control diet + NCG (40 mg/kg BW) vs. urea diet vs. urea diet + NCG (40 mg/kg BW)	Mixed in feed	2-week diet adaptation 7-week treatment (2 times daily feeding)	Control vs. Control + NCG: ↓ Faecal N (8.2 vs. 69.9 g/d) ↓ Urinary N (105 vs. 95.2 g/d) ↑ N retention (62.2 vs. 73.3 g/d) ↑ N utilization (0.24 vs. 0.31 g/d) ↓ BUN (3.52 vs. 3.14 mmol/L) ↓ Plasma ammonia (55.1 vs. 45.5 μmol/L)	[76]

The concentration of MUN has been used as an indicator of the amount of UN excreted [81], and the reductions in PUN and MUN suggest that supplementation with NCG could lead to reductions in UN output. Supplementation with NCG has resulted in quadratic decreases in the concentration of UN in lactating cows, with the lowest concentrations observed when NCG was fed at ~ 30 mg/kg BW. A similar quadratic response in UN output, using urinary creatinine as a marker for urinary volume voided, was reported by Yang et al. [75] for fattening bulls, with the lowest UN output when NCG was provided at 40 mg/kg BW (a 15% reduction relative to the unsupplemented control). Similarly, Zhang et al. [73] reported reductions in UN output when NCG was supplemented (40 mg/kg BW) to fattening bulls, regardless of the source of protein in the diet (soybean mean or urea). In the latter study, urinary N excretion was estimated to be 10% and 15% less than that from unsupplemented control diets containing soybean meal and urea, respectively. Faecal N (FN) is also a source of environmental pollution, particularly for housed systems where manure is gathered and stored. In grazing systems, FN deposited on grazed pasture contributes to nitrous oxide and nitrate issues, albeit at a smaller scale than urine. Two studies with fattening bulls indicate the potential of NCG to reduce this N pollution source. Yang et al. [75] reported a trend for NCG to reduce FN, with a dose of ~ 30 mg/kg causing the greatest numerical reduction, while [73] reported a ~ 15% reduction in FN when NCG was supplemented to diets containing either soybean meal or urea as protein sources.

Excess N intake and dietary CP concentration are two main drivers of PUN and MUN concentration and, ultimately, UN excretion [81, 82]. The reductions in UN and FN excretion discussed above and presented in Table 4, have been obtained with diets formulated to meet the CP requirements of the cows and bulls in the studies (e.g., 13%–18% CP). Thus, the magnitude of the reductions in UN and FN are in line with the additional protein deposition in milk and meat when dietary protein supply is close to the animal requirements. In conditions of dietary N excess, the potential for an increased proportion of N intake being retained in milk may be smaller. The effects of NCG when ruminants are fed protein-rich diets, such as those used in intensively managed temperate grazed pasture would therefore need to be established.

While the reduction in UN and FN output is most desirable, potential exists for NCG to provide a tool for management of the urine patch in grazing systems [83] if the 15%–25% reduction in UN concentration reported by Chacher et al. [39] is a consistent effect of this additive. More studies are required to assess if UN concentration is consistently reduced when animals are offered grazed forages.

In temperate grazing systems, feeding crops with low N content, such as fodder beet, has been recommended as a practice to mitigate UN excretion to the environment. However, reports suggest that the productive potential of animals fed this crop is not fully realised [84, 85]. Feeding fodder beet at 23% to 45% of the DM reduced circulating Arg concentrations in lactating dairy cows [79], leading these authors to postulate that reduction in the supply of specific AA such as Arg could impact the potential of fodder beet as a tool to manage N intake in temperate dairy systems. An increase in circulating concentrations of Arg is a common response to NCG supplementation [39, 40, 42, 45], suggesting a potential for NCG to be a complementary strategy to enable the use of low-N crops as tools to mitigate N excretion in temperate grazing systems. However, this hypothesis would require evaluation with animal studies.

There is evidence suggesting the potential of NCG as a tool to mitigate the environmental impact caused by N excretion, both UN and FN, when diets are formulated to meet the CP requirements of different species and classes of ruminants. However, the effects of NCG supplementation to ruminant livestock fed fresh forage diets that are high in both protein and protein degradability (e.g., temperate grazed pasture) still needs to be determined directly, preferably using total collection of excreta to confirm the findings reported in the literature.

### Summary

**Fig. 2:**
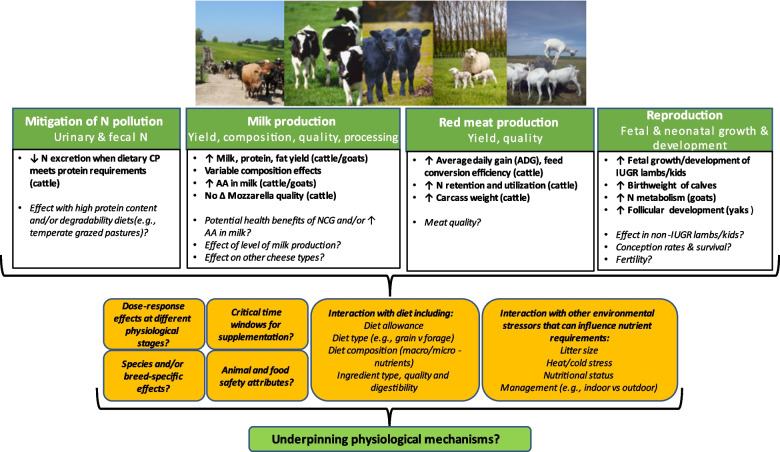
An overview of the current knowledge of the impacts of N-carbamoylglutamate on the mitigation of nitrogen (N) pollution, milk and red meat production and reproduction in ruminants

Ruminant livestock production of meat, milk, fibre and co-products play a key role in human wellbeing globally. Improving N metabolism in ruminants benefits livestock production efficiency and reduces environmental N pollution via urine and faeces, particularly in pasture-based production systems. New technologies such as dietary supplementation with NCG, may provide an opportunity to improve the efficiency of livestock production and animal welfare through improved survival, growth, lactation and reproductive performance while simultaneously reducing environmental footprint through improved N utilization efficiency. There is a growing body of evidence from ruminant (cattle, sheep and goats) studies in China suggesting potential benefits of dietary supplementation with NCG. However, further studies are required to determine critical physiological time windows for administration, acute vs*.* chronic effects, dose–response effects, species-, breed- and/or sex-specific responses, and management systems (e.g., grazing vs*.* non-grazing, indoor vs. outdoor etc.) on animal responses (Fig. 2). The influence of the composition and allowance of the basal diet (e.g., grain vs. forage, macro- and micro-nutrient profiles etc.) also needs to be evaluated, particularly relevant for pasture-based production systems where the N content of forages is often substantially greater than TMR or grain-based rations. Nevertheless, the growing body of literature in ruminants for NCG to act as a functional nutrient to improve production capacity and N utilization efficiency highlights the potential for commercial application.

## Data Availability

Not applicable.

## Abbreviations

AA: Amino acid
ADG: Average daily gain
Arg: Arginine
BW: Body weight
CP: Crude protein
CPS-1: Carbamoyl-phosphate synthetase-1
DIM: Days in milk
DMI: Dry matter intake
IGF-1: Insulin-like growth factor 1
IUGR: Intra-uterine growth restriction
mTOR: Mammalian target of rapamycin
MUN: Milk urea nitrogen
N: Nitrogen
NAG: N-acetyl glutamate
NCG: N-carbamoylglutamate
NEAA: Non-essential amino acid
NO: Nitric oxide
NPN: Non-protein nitrogen
NRC: National Research Council
PUN: Plasma urea nitrogen
SCC: Somatic cell count
TMR: Total mixed ration
UN: Urinary nitrogen
